# Temperature-dependence of life history in an edible cricket: Implications for optimising mass-rearing

**DOI:** 10.1016/j.cris.2025.100109

**Published:** 2025-03-10

**Authors:** Jacinta D. Kong, Émile Vadboncoeur, Susan M. Bertram, Heath A. MacMillan

**Affiliations:** Department of Biology, Carleton University, Ottawa, Ontario K1S 5B6, Canada

**Keywords:** Insect rearing, Insect agriculture, Thermal performance, Alternative protein production

## Abstract

Optimisation of life history and organismal performance underlies success in insect mass-rearing. Rearing schedules, resource use and production yield depend on many aspects of insect fitness and performance within and across generations, such as growth, development, longevity, and fecundity, which are all temperature dependent. Despite this general understanding, we often lack species-specific information needed to make informed decisions about manipulating rearing temperatures to optimise insect growth and development. Here, we characterise the effects of rearing temperature on nymph to adult development and lifespan (20 – 38 °C), and reproductive output (30 – 38 °C) in a farmed cricket (*Gryllodes sigillatus*). Crickets grew larger and reached adulthood sooner at higher developmental temperatures at the expense of longevity. Reproductive output was similar across a range of temperatures but decreased at 38 °C. Therefore, while temperature control is necessary to maximise production rates, temperature is unlikely to affect production yield in a fixed harvest cycle provided it is maintained within the narrow range enabling both fast growth and stable reproduction (32 – 36 °C). Our study provides a fundamental basis for further optimisation of insect rearing operations and a deeper understanding of the thermal biology of this commonly farmed species.

## Highlights

• Rearing temperature (20–38 °C) had substantial effects on life history traits in an edible cricket.
• Growth, development and reproduction defined a range of temperatures suitable for mass-rearing (30–36 °C).
• Yield estimates revealed times and temperatures that maximize yield for harvest.
• The data are a first-step towards optimizing and evaluating industrial mass-rearing practices.

## 1 Introduction

Alternative sources of protein, such as insects, are a promising solution to the growing problem of feeding an increasing global population (van Huis, 2013; UN, 2015). Insect farming achieves the requirements for meeting global food security by being space-efficient, scalable and able to be integrated within sustainable practices, such as a circular food economy (Chavez, 2021; Liceaga et al., 2022). Rising interest in insect farming globally has driven rapid growth in an increasingly competitive market where the challenges for individual operators are to increase their output (yield) to meet changing market demands and attain economic sustainability (Tanga et al., 2021; Larouche et al., 2023). The simplest estimation of yield is biomass. For a fixed area farmed, increasing the total mass of a farmed insect population at harvest can be achieved by targeting life history traits of the farmed species. Relevant traits include increasing adult mass, development rate, survival, and fecundity. Adult mass and survival of the population will affect the total biomass harvested at the end of the production cycle (Eggink et al., 2023). Development rate can influence the timing of the harvest (and thus yield per unit time) if the production cycle is flexible to when adults are present in the population and whether the population is also used for breeding (Lehtovaara et al., 2018). Fecundity is important to the continued production of the farm (Ormanoglu et al., 2024). All these life history traits are influenced by temperature as the effect of temperature on the rates of all enzymatic reactions are critical for ectothermic species such as insects (Kingsolver and Buckley, 2020). We can therefore use thermal plasticity of insect life history to identify the range of temperatures that produce the desired trait values or a single optimal temperature for mass-production (Chia et al., 2018). Collecting, analysing and applying this life history data to insect farming will allow for data-driven management decisions (Davidowitz, 2021).

To leverage thermal plasticity for insect farming, we must consider two important caveats: first, the thermal plasticity of traits measured under constant temperatures are typically non-linear following a thermal performance curve (Raynaud-Berton et al., 2024). The magnitude and directionality of this plasticity in performance may vary among traits such that the optimal temperature for one trait does not match other traits (Kellermann et al., 2019). Nevertheless, positive synergies among traits may emerge. For example, development rate may be more thermally plastic than survival, except at extreme temperatures (Kingsolver and Buckley, 2020). This synergy may allow an insect farm to take advantage of a higher temperature without incurring costs to survival and yield. Second, traits often trade-off in a manner that reflects evolutionary relationships with temperature (Tüzün and Stoks, 2022). For example, higher rearing temperatures of *Hermetia illucens* allow for faster growth to adulthood but negatively impact reproduction and longevity, which consequently defines the optimal temperature range for mass-rearing (Chia et al., 2018). It is thus important to ensure these life history trade-offs do not affect yield in the continuous rearing of insects for farming. Characterising thermal performance of relevant traits for a given species across the entire range of temperatures relevant to life history can capture the limits of performance and identify trade-offs, but for practitioners this data may be unavailable or challenging to collect with limited resources (Davidowitz, 2021; Larouche et al., 2023).

The decorated cricket, *Gryllodes sigillatus* (Orthoptera: Gryllidae), is farmed for animal feed production and human consumption (Larouche et al., 2023). Several studies have described aspects of life history for this species under controlled temperature conditions (Sakaluk et al., 2019; Muzzatti et al., 2022; Kong et al., 2024). However, too few descriptions of *G. sigillatus* thermal biology are available to make informed management decisions or predictions of growth and development related to environmental temperatures for mass-production. To fill this knowledge gap, we first aimed to characterise the effect of rearing temperature on cricket growth, development and reproduction across the life cycle. We hypothesised that temperature affects the life history of decorated crickets through four fitness components: 1) higher temperatures result in smaller adult body sizes (Verberk *et al*. 2021), 2) development to adulthood is faster at higher temperatures (Lehtovaara et al., 2018), 3) survival decreases at higher temperatures (Andreadis et al., 2014), and 4) fecundity increases with increasing temperatures far below the limits of survival and decreases at temperatures approaching the limits of survival (Chia et al., 2018). To test our hypothesis and its four predictions, we reared crickets from hatch to adulthood and death at 10 temperatures between 20 and 38 °C (in 2 °C increments). Using our results, we next identified optimal temperature ranges for mass production. We then combined outcomes for the life history traits to calculate an index of yield that we used to evaluate the temperatures and harvest times with the potential to maximise yield. From this fundamental understanding of temperature-dependent performance of this species, we identified temperatures that optimise increases in mass and decreases in development time, as well as potential trade-offs in survival and reproduction across the life cycle that ultimately determine yield. The potential effects of rearing temperature can be evaluated against the potential operative costs associated with rearing temperature, such as feed consumption and heating.

## 2 Methods

### 2.1 Growth, development, survival & longevity

Eggs were obtained from Entomo Farms, Norwood, Ontario, Canada that were laid in moist coco peat at the farm and shipped to Carleton University, Ontario, Canada. The eggs were incubated at 32 °C 14 h:10 h L:D photoperiod (Isotemp refrigerated incubator, Fischer Scientific, Ontario, Canada) to complete egg development in the laboratory. Hatchlings that emerged within 24 h were individually weighed on an analytical balance (Mettler Toledo AB135-S, Ontario, Canada). They were then individually housed in a small container (96 mL, 7 cm diameter × 3 cm depth) and provided with a piece of cardboard egg carton for shelter. Crickets were provided fresh food as a mix of corn, soy, fishmeal and micronutrients (Campbellford Farm Supply, Ontario, Canada) once a week in a vial lid (1.7 cm diameter × 1.4 cm depth) glued on a 5 cm petri dish. Water was provided three times a week using a lidless 1.5 mL microcentrifuge tube capped with dental cotton. Containers (*n* = 24 at each temperature) were incubated in a reptile egg incubator (XHC-25, Vevor Inc., Cucamonga, USA) at one of 10 temperature treatments (20 – 38 °C, in 2 °C increments, 60–80% relative humidity) for a total of 240 crickets. A datalogger recorded temperature and humidity every 30 min (IBS-TH2, Inkbird, Shenzhen, China). Crickets that died in the first three days of incubation were replaced with individuals from the same cohort of eggs (*n* = 7) and subsequent deaths were not replaced. Mass and instar were recorded for each cricket weekly until death or 27 weeks since hatch. Sex was recorded when sex could be easily determined between instar 6–7 and at adulthood (Kong et al., 2024). After 27 weeks the surviving crickets were adults (based on the development of wings in males and ovipositors in females) and were maintained until death. Changes in instar were noted every three days during routine maintenance when crickets were at sixth instar and above. Crickets incubated at 20 and 22 °C were culled after 8 weeks because growth and development were very poor and no crickets had reached sixth instar (indicating these temperatures were far from the optimum).

**Fig. 1:**
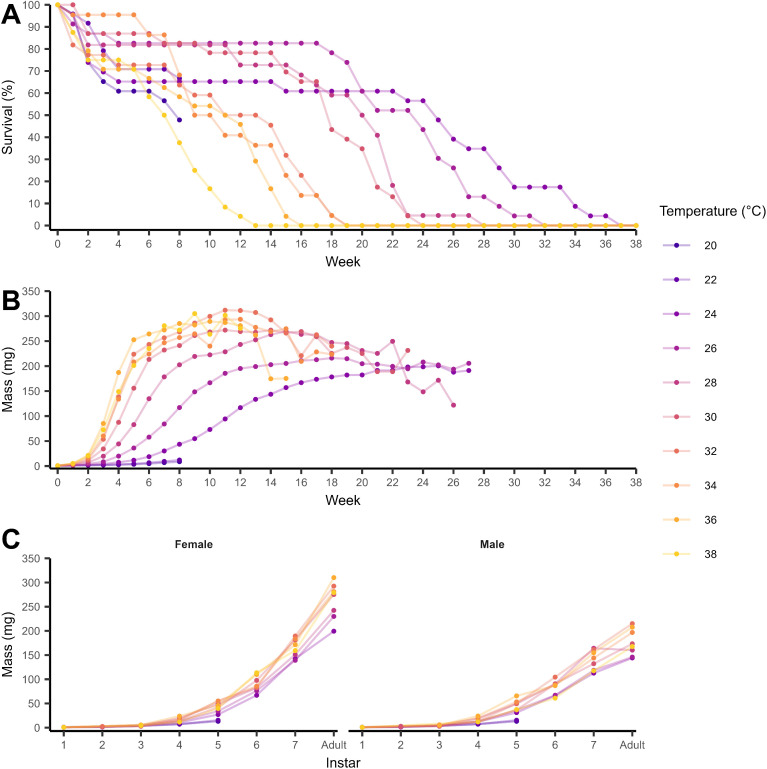
A) Percentage survival of *Gryllodes sigillatus* at the start of each week until all individuals had died. B) Weekly mean mass (mg) Weekly measurements of mass were stopped at 27 weeks. Error bars around the mean are not shown C) Mean mass (mg) at each nymphal instar and at adulthood. Crickets (*n* = 22 – 24 crickets per temperature) were reared at 10 temperatures between 20 and 38 °C (colours). Crickets were maintained from hatching (24 h old) until death except for crickets at 20 and 22 °C which were culled after 8 weeks (shown in both panels in 1C because sex could not yet be determined).

A total of 183 crickets were used for analysis from a total of 247 crickets. Crickets that died in the first three days or had accidental deaths (*n* = 17), and crickets reared at 20 (*n* = 23) and 22 °C (*n* = 24) were excluded. Crickets reared at 20 and 22 °C are still shown in Fig. 1. The temperature-dependence of adult mass, development rate to adulthood, survival to adulthood, and longevity was assessed. To account for changes in adult mass over time, adult mass for each cricket was calculated as the mean of the first four weekly observations of mass when crickets were adults (79 females and 62 males). Development time to adulthood was calculated from the start of the experiment (24 h old crickets) to the date of moulting to adulthood. Development rate to adulthood was calculated as the inverse of development time. Longevity was calculated for crickets at 24 – 38 °C from the start of the experiment until death. Adult mass, development rate and longevity were checked to meet the assumptions of normality, then analysed using linear regression in R v4.3 with temperature as a categorical variable and using 32 °C as the baseline contrast (R Core Team, 2024). The baseline contrast was set to 32 °C because that is the standard rearing temperature for *G. sigillatus* in a laboratory (Archer et al., 2012). An initial analysis of a full model with temperature and sex, and their interaction did not find the effect of temperature to be dependent on sex for adult mass (Temperature × Sex: F_7, 125_ = 0.57, *P* = 0.78), development rate (Temperature × Sex: F_7, 125_ = 1.20, *P* = 0.31), or longevity (Temperature × Sex: F_7, 128_ = 0.66, *P* = 0.71). These interaction terms were dropped from these models. Tukey's Honest Significant Differences were used as post-hoc tests. We used a Chi-squared test to evaluate the proportion of crickets surviving to adulthood among temperatures. All variances around the mean are shown as standard error.

### 2.2 Maximum potential yield

An individual-level yield index calculated as yield index = mass/time was used to identify temperatures and times at which potential yield can be maximised. Adult mass and development time to adulthood were used to assess maximum potential yield per individual at adulthood. Weekly mass and week were used to determine the optimal harvest timepoint for maximal potential yield between weeks 0–7. Potential yield (mg day^−1^) for each incubation temperature was calculated as the mean yield index for all individuals at that temperature. Survival was not included because survival is measured at the population level. Potential yield at adulthood was analysed using linear regression with an interaction between week and temperature. Tukey's Honest Significant Differences were used as post-hoc tests. Potential yield between weeks 0–7 were not statistically assessed.

### 2.3 Reproductive output

Eggs from a colony of *G. sigillatus* (originally derived from the same Entomo Farms population) maintained at Carleton University, Ontario, Canada were collected to characterise the effect of temperature on fecundity (detailed in Kong et al. 2024). We used an independent cohort of crickets to avoid confounding potential effects of reproductive effort on longevity. Eggs were collected in moist peat for 24 h then incubated at 32 °C 14L:10D photoperiod (Isotemp refrigerated incubator, Fisher Scientific, Ontario, Canada) with food and water to complete development and emerge. First instar crickets were allocated to one of 5 temperature treatments (30, 32, 34, 36 or 38 °C, 14L:10D photoperiod, inside 10 reptile egg incubators, (XHC-25, Vevor Inc., Cucamonga, USA) within 24 h of emergence for a total of 200 crickets (20 crickets per incubator, 2 incubators per temperature). Since we found development was slow at temperatures below 30 °C, we focused on a narrower, farming-relevant, range of temperatures that allowed us to rear to adulthood and test for trade-offs in reproductive output. Parental crickets were housed individually and maintained as described above. Survival, sex and instar were recorded three times a week during maintenance following Kong et al. (2024).

Crickets at the same incubation temperature that eclosed within the same week were allocated as mating pairs to control for age. Adult crickets were weighed on an analytical balance (Mettler Toledo AB135-S, Ontario, Canada) then assigned a mating pair. The number of mating pairs (*n* = 70) depended on the number of crickets surviving to adulthood (Table 1). Three pairs that died within eight days without producing eggs were excluded, resulting in 67 pairs for analysis. Mating pairs were set up at 32 °C 14L:10D (Isotemp refrigerated incubator, Fisher Scientific, Ontario, Canada) in a container (10 × 16 × 6 cm) with feed, water, shelter and oviposition substrate (water and marbles in a 96 ml container). The same mating pairs were used throughout, but crickets were not acoustically isolated from other pairs (Bateman and MacFadyen, 1999). Egg collection followed methods of Kong et al. (2024). The oviposition substrate, water and marbles, was replaced every other day from the time pairs were formed.

**Table 1:** Survival to adulthood, offspring production, and hatching success of *Gryllodes sigillatus* reared at five temperatures between 30 – 38 °C. Mating pairs (*n* = 67) were maintained at 32 °C.

Rearing temperature (°C)	Percentage survival to adulthood (starting number of crickets)	Number of mating pairs with eggs incubated (number of pairs)	Mean offspring production ± standard deviation (number of nymphs per pair)	Mean hatching success (% eggs hatched per pair)
30	91.9 (37)	11 (15)	10.7 ± 8.54	18.0
32	94.4 (36)	11 (14)	19.7 ± 22.3	26.4
34	94.7 (38)	12 (17)	27.7 ± 29.4	31.9
36	89.7 (39)	10 (16)	13.3 ± 18.4	21.9
38	30.0 (40)	3 (5)	0 ± 0	0

We characterised reproduction in two ways: 1) oviposition delay, defined as the time between when mating pairs were introduced to when the first egg was oviposited, and 2) fecundity, as mean fecundity (mean number of eggs oviposited per 48 h) and as total fecundity (total number of eggs oviposited during the study period). To account for very productive pairs and delays in maturation, we time-censored egg production between when oviposition started and the date when the following criteria were met: 1) pairs had laid seven batches of five or more eggs, 2) the female died or, 3) when the experiment was stopped after 54 days. We stopped recording egg production after 54 days because most pairs had laid sufficient eggs by this time point. Crickets were culled after one of these conditions were met. Thus, the data do not capture lifetime fecundity. We used all observations within this censored time frame to calculate fecundity regardless of the number of eggs oviposited per batch, resulting in 1–19 observations per pair including observations of zero eggs. A batch was defined as five or more eggs as a conservative independent sub-replicate because we could not determine if a small number of eggs (less than five) oviposited on a given timepoint was independent from the batch oviposited at the previous timepoint. Seven sequential batches of eggs represented approximately 14 days of oviposition after which oviposition is consistent based on Kong et al. 2024. Batches 5–7 which represented peak oviposition were incubated to measure hatching success and egg development time. All eggs in a batch were transferred to moist peat and incubated at 32 °C 14L:10D (Isotemp refrigerated incubator, Fisher Scientific, Ontario, Canada). We incubated at least one batch of eggs from 47 pairs but not all pairs produced sufficient batches. All other batches of eggs were recorded and discarded. The batches of eggs that were set aside to incubate were checked daily for the first day of hatch. Hatching time for each batch of eggs was taken as the day the first nymph was observed. Two days after first emergence, the containers with peat and newly hatched nymphs were frozen for 24 h, then dried in an oven at 60 °C for a minimum of five days (4EM Incubator, Precision Scientific, Illinois, U. S. A). The dried nymphs were counted to calculate offspring production per replicate pair (mean number of offspring per pair), as well as hatching success from the number of dried nymphs and the number of eggs incubated. We used mean fecundity and mean hatching time for each pair as the level of replication. Pairs that failed to oviposit any eggs (fecundity of 0, *n* = 4) and pairs that oviposited batches of less than five eggs (*n* = 4) were included in the analysis. Oviposition delay (63 pairs), fecundity (67 pairs) and hatching time (40 pairs) were analysed using linear regression with temperature as a categorial variable. To evaluate the effect of parental size on fecundity, we tested the effect of 1) female mass, 2) male mass, and 3) the ratio between female and male mass on fecundity.

## 3 Results

### 3.1 Growth, development, survival & longevity

**Fig. 2:**
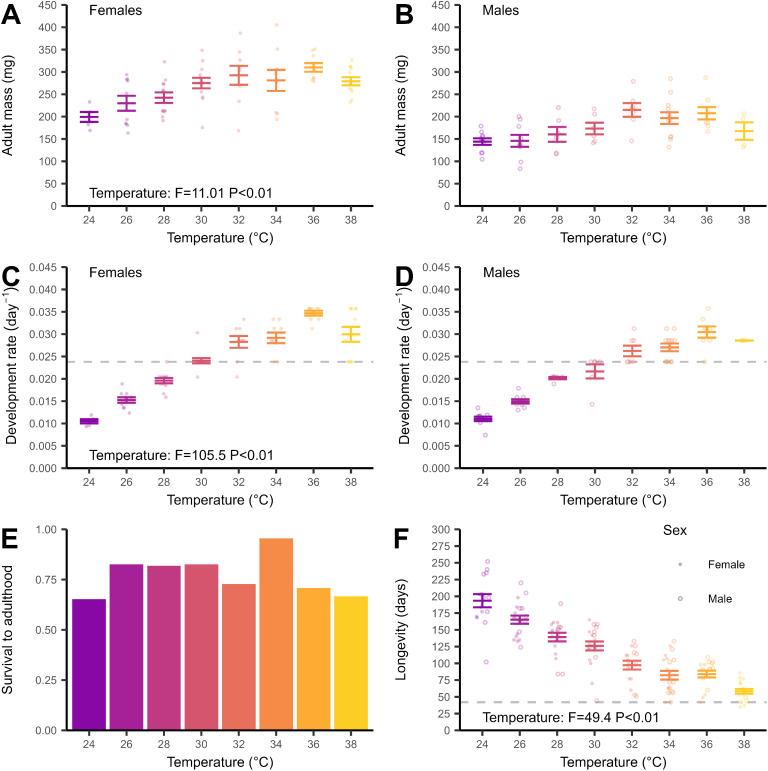
Life history traits for female (closed circles) and male (open circles) *Gryllodes sigillatus* crickets reared at one of 8 incubation temperatures between 24 – 38 °C (colours). Adult mass (mg, points) for 79 females (A) and 62 males (B). Development rate to adulthood (day^−1^, points) for 79 females (C) and 62 males (D). Development rate was calculated as the inverse of development time (time to reach adulthood since the start of the experiment). Proportion of crickets surviving to adulthood at each incubation temperature (E), and (F) longevity (days, points) from hatching to death for 81 females and 62 males. Grey dashed line indicates a 6-week harvest cycle as a benchmark. Error bars are standard error about the mean (middle line).

Survival decreased over time and with steeper declines at higher temperatures as crickets reached adulthood (Fig. 1A). All crickets reared at temperatures 24 °C and above had died by week 37 (Fig. 1A). The growth pattern of *G. sigillatus* followed a sigmoidal pattern over time (Fig. 1B). Decreases in mass after 14 weeks for temperatures above 30 °C were associated with the increasing mortality of the cohort as they aged (See Fig. 1A). Mass at each instar increased in an exponential pattern (Fig. 1C). Mean cricket mass ranged from 0.73 ± 0.01 mg for first instar crickets at the start of the experiment, including at 20 and 22 °C, to 217.90 ± 1.37 mg for adult crickets. Adult females were heavier than males (F_1, 132_ = 133.88, *P* < 0.001) with a mean of 258.87 ± 1.55 mg for adult females (Fig. 2A) and 170.04 ± 1.30 mg for adult males (Fig. 2B). There was an overall effect of temperature on mean adult mass (F_7, 132_ = 11.01, *P* < 0.001) with heavier crickets at 30 °C and above. Mean adult mass was similar across temperatures above 30 °C (Tukey's HSD, *P* > 0.05).

Development rate varied among temperatures (F_7, 132_ = 105.52, *P* < 0.001, Fig. 2C& D) with faster development at higher temperatures excepting a decrease in development rate at 38 °C, but this decrease was modest and development rate at 38 °C was similar to 32 and 34 °C (Tukey HSD, *P* > 0.05). Crickets reared at 36 °C reached adulthood the fastest on average (31.5 ± 0.3 days) and crickets at 24 °C developed the slowest to adulthood (94.1 ± 0.7 days). Females reached adulthood sooner than males on average (51.0 days ± 0.6 for females, Fig. 2C; 58.7 ± 0.7 days for males, Fig. 2D, F_1, 132_ = 7.91, *P* = 0.006).

Survival to adulthood averaged 77.2% across 24 – 38 °C with 141 of 183 crickets surviving to adulthood (79 females and 62 males). Survival to adulthood did not differ between 24 – 38 °C (Fig. 2E, χ^2^ = 9.3, d.f. = 7, *P* = 0.23). Longevity varied among crickets reared between 24 – 38 °C (F_7, 135_ = 49.42, *P* < 0.001) with crickets at higher temperatures having shorter lifespans than at lower temperatures (Fig. 2F). Minimum lifespan was 35 days for females at 38 °C and 42 days for males at 34 °C. Maximum lifespan was 203 days for females and 252 days for males at 24 °C. Mean longevity did not differ between the sexes across temperatures (81 females and 62 males, Fig. 2F, F_1, 135_ = 1.27, *P* = 0.26). When crickets reared at 20 and 22 °C were culled at the end of eight weeks, survival was 47.8 % and 66.7 % for crickets at 20 and 22 °C, respectively (Fig. 1A), mass was 8.67 ± 1.54 mg and 12.04 ± 1.62 mg for crickets at 20 and 22 °C, respectively (mean ± standard error, Fig. 1B), median instar was 4 for both temperatures with a range of 2–5 for crickets reared at 20 °C, and 3–5 for crickets reared at 22 °C.

### 3.2 Maximum potential yield

**Fig. 3:**
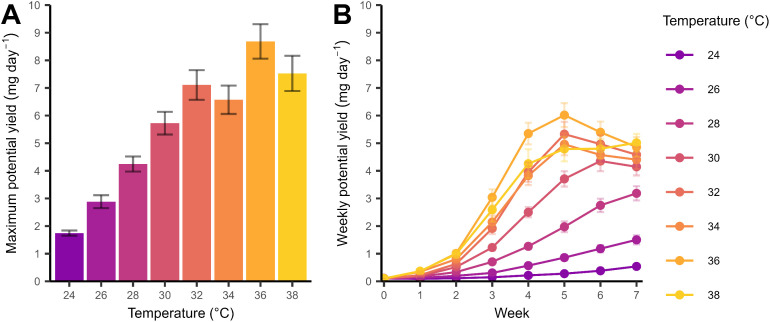
Potential yield (mg day^−1^) calculated A) at the onset of adulthood (maximum potential yield) and B) from weekly mass each week up to week 7 (weekly potential yield). The benchmark production cycle is at 6 weeks. Points are means for individuals at a rearing temperature (colours). Error bars show standard error around the mean.

Maximum potential yield increased with temperature if crickets were harvested at the onset of adulthood with a maximum yield of 8.68 mg day^−1^ per individual cricket reared at 36 °C (Fig. 3A, F_7, 133_ = 26.4, *P* < 0.001). Maximum potential yield from crickets reared at 36 °C was similar to crickets reared at 32 and 38 °C (Tukey HSD, *P* > 0.05). Weekly potential yield for rearing temperatures 30–36 °C increased to a maximum at week 5, with a maximum yield of 6.01 mg day^−1^ for crickets reared at 36 °C, then declined at weeks 6 and 7 (Fig. 3B). Potential yield for crickets reared at 38 °C increases only marginally between weeks 5 and 7. Maximum yield is delayed to increasingly later timepoints for rearing temperatures 28 °C and below (Fig. 3B).

### 3.3 Reproductive output

**Fig. 4:**
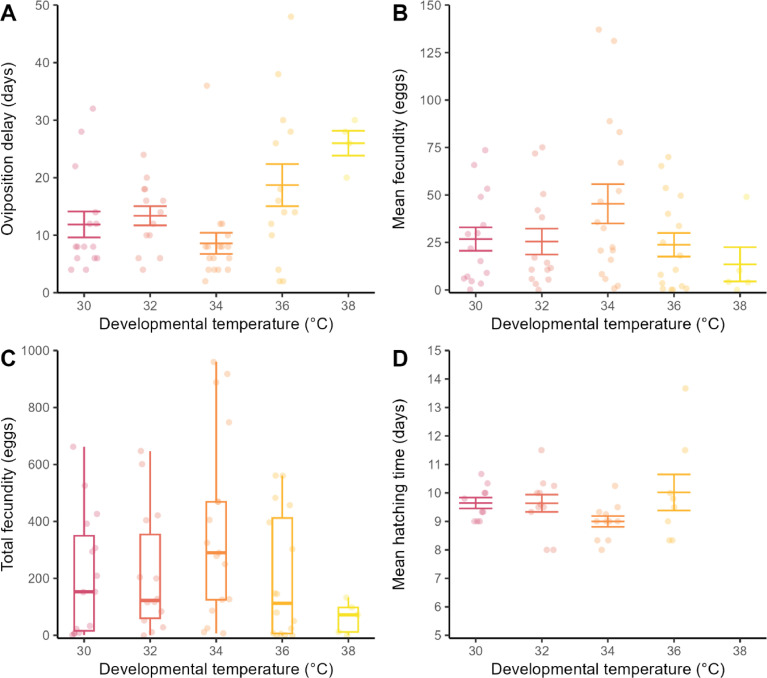
A) Delay in first oviposition (days, points) since *Gryllodes sigillatus* crickets were paired (*n* = 63), B) mean number of eggs oviposited every 48 h per pair (*n* = 67, points), C) total fecundity of each pair (*n* = 67, points) for the sampling period and box and whiskers plots, and D) mean hatching time (days, points) for the first nymph per batch to emerge per pair (*n* = 40). Crickets were reared between 30 and 38 °C (colours) until adulthood then allocated to mating pairs that were maintained at 32 °C for oviposition and egg incubation. Error bars are standard error about the mean value for each temperature (middle line).

Four pairs did not produce eggs after at least 10 days, giving 63 pairs that produced at least one egg (Table 1). Rearing temperature affected the delay in oviposition (Fig. 4A, F_4, 58_ = 4.28, *P* = 0.004), but did not affect either the fecundity of a pair (Fig. 4B, F_4, 62_ = 1.8, *P* = 0.15) nor hatching time (Fig. 4D, F_3, 36_ = 1.58, *P* = 0.21). The total number of eggs ranged between 0 and 960 eggs per pair (median 145 eggs, Fig. 4C). The number of eggs laid in 48 h was also highly variable, ranging between 0 and 297 eggs. Oviposition delay was similar between 36 and 38 °C (Tukey HSD, *P* > 0.05) and both were different to 34 °C (Tukey HSD, *P* < 0.05) but not 32 and 30 °C. Crickets reared at 38 °C had the longest mean delay in first oviposition, the lowest mean fecundity, and none of the incubated eggs hatched (Fig. 4). Crickets reared at 34 °C had the shortest mean oviposition delay, the highest mean fecundity, and consequently, the highest reproductive effort (Table 1, Fig. 4). Fecundity increased with increasing female mass (slope = 0.16, t_61_ = 2.4, *P* = 0.018) and with the ratio of female to male mass (slope = 20.2, t_61_ = 2.0, *P* = 0.047), but did not depend on male mass (slope = −0.01, t_61_ = −0.1, *P* = 0.92).

## 4 Discussion

Life history drives insect population growth and biomass and insect life history is directly influenced by temperature (Meister et al., 2017; Kingsolver and Buckley, 2020). Decorated crickets have a tropical distribution that suggests they are adapted to warm climates (Toms, 1993). Indeed, we found the thermal sensitivity of *G. sigillatus* skews heavily towards higher temperatures within the range of temperatures we investigated (20–38 °C). Crickets reared at 30 °C and above were heavier, grew faster but had a shorter lifespan, and reproductive output declined at 38 °C. In contrast, crickets reared at 20 and 22 °C performed poorly in development, growth and survival, and much more so than crickets reared at 24 °C (Fig. 1). While smaller body sizes at higher temperatures are reported for crickets and ectotherms in general, the opposite trend we observed aligns with global patterns of temperatures and traits across many terrestrial tropical insects (Kingsolver and Huey, 2008; Abarca et al., 2024; Booth and Kiddell, 2007). Our results demonstrated *G. sigillatus* is highly amenable to temperature manipulations and to high temperature rearing within the limits of their fitness. Our data provides a first step towards more targeted optimisation problems at an industrial scale based on biological information.

Development rate is thought to influence plasticity in size with temperature (Walters and Hassall, 2006). The difference in development time to adulthood at 32 °C (36.3 days for females, 38.5 days for males) was comparable to the development times reported in Sakaluk et al. (2019) for outbred *G. sigillatus* (36 days for females and 39 days for males reared at 31 °C). It is possible that our observation schedule of 3–4 days was too large to capture fine-scale inter-individual variation in the timing of development, especially at the higher temperatures. An absence of interaction between sex and temperature in our data suggests thermal plasticity does not affect the sexual size dimorphism in this species. We found the plasticity in developmental rate does not explain the modest increases of adult mass with temperature (compare Figs. 2C & D with A & B). This pattern suggests development rate and adult mass are evolutionarily decoupled, which could reflect a constraint on adult mass resulting in canalisation at temperatures above 30 °C. Proposed explanations for a canalisation of mass include an energy imbalance allocating energy away from biomass accumulation, differential increases in length among body parts (McDonald et al., 2018), or a limit to body size set by the physiological demands of a larger size, such as the enlargement of air sacs to support larger insects (Harrison et al., 2023). Despite the small magnitude of plasticity in adult mass, we observed males and females can vary greatly in mass at the same rearing temperature (Figs. 2A & B, Coefficient of Variation ranging between 9.4 – 25 % in females and 16 – 27 % in males). Future work can investigate how variation in adult mass is generated from the same cohort under the same rearing temperature, and the importance of sex in how growth patterns emerge throughout ontogeny as our data suggests differences in mass are most apparent at instars when sex characteristics appear (Fig. 1C). We had insufficient numbers of each sex at each temperature to formally investigate within sex differences in life history but the magnitude of sex-specific differences in the life history traits we measured may not be large (Teder et al., 2022).

Longevity from hatching to death has not been previously reported for *G. sigillatus* but our longevity at the adult stage was within the ranges previously reported for *G. sigillatus* (Archer et al., 2012). Crickets reared at 32 °C in our study lived 63.1 ± 1.7 days for females and 70.0 ± 2.6 days for males after reaching adulthood, compared with 50.4 days for females and 73.6 days in males reported for outbred lines reared at 32 °C (Archer et al., 2012). Reduced lifespans at higher temperatures are directly relevant to mass-rearing if crickets die sooner than a benchmark harvest time. As most crickets survived longer than the six-week benchmark harvest time considered here except for a few individuals between 34 and 38 °C (points below the dashed line, Fig. 2F), the temperatures we considered may not be detrimental for mass-rearing. However, the temperature-longevity relationship can indirectly affect farming operations through its association with faster ageing and senescence, which affects fecundity and consequently, reproductive output for ensuring the next generation (Kelly et al., 2013; Kindlmann et al., 2001). Therefore, the potential for negative effects on longevity and reproduction at higher temperatures (e.g. 38 °C) may outweigh any benefit of these temperatures in other life history traits, unless the timing of the production cycle is adjusted accordingly. The thermal plasticity in longevity did not appear to be strongly determined by survival bias during nymphal development as crickets reared at temperatures with similar survival to adulthood have different longevities (Fig. 1).

We found reproduction under common garden temperatures at 32 °C was similar for crickets reared to adulthood between 30 and 36 °C (Fig. 4). This pattern could either indicate carry-over effects of temperature during nymphal development were minimal within these rearing temperatures or that crickets compensated for developmental temperature (Moore and Martin, 2019). Regardless, the strong decrease in reproductive output for crickets reared at 38 °C indicated a threshold response of reproduction to temperature that is sensitive to nymphal development and is expressed after relocation to lower temperatures. Thus, 38 °C is likely the limit of reproduction and, because reproduction is an important component of fitness, of rearing even if crickets can survive to adulthood above this temperature. Reproductive traits are more thermally sensitive to temperature with lower thermal limits than other traits, such as survival (Walsh et al., 2019). Decreased fecundity at 32 °C for crickets reared at 38 °C was primarily driven by decreased oviposition expressed as both a delay in the start of oviposition, in the rate of oviposition and in the number of eggs oviposited. Fecundity in our study (number of eggs in 48 h) was lower than the 151 eggs in 48 h reported for outbred *G. sigillatus* 10 days post-eclosion to adulthood in Archer et al. (2012), and the ∼125 eggs for the same duration in Kong et al. (2024). Delays in oviposition in this study were longer than 4–6 days reported previously (Bateman et al., 2005; Kong et al., 2024). The increased delay in oviposition for crickets reared at 38 °C could indicate that crickets are maturing later while under common garden conditions. For example, one pair reared at 38 °C produced two large batches of eggs (> 25 eggs) after 26 days then stopped ovipositing for the remainder of the experiment. Decreases in fecundity or a delay in maturation could reflect energy use throughout development. If crickets are prioritising energy towards maintenance at high temperatures at the cost of maturation, then reproductive organs could be underdeveloped or smaller relative to lower temperatures, as observed in the yellow dung fly (*Scatophaga stercoraria*) and the butterfly *Bicyclus anynana* (Blanckenhorn and Henseler, 2005; Steigenga and Fischer, 2007). The failure to hatch for eggs from 38 °C reared crickets may stem from several components of fertility in either sex. Oviposition was possible because most of the females (4/5) at this temperature oviposited at least one egg. These eggs could have been unfertilised if sperm form and function were affected or if mating was not successful, but we did not record mating behaviours (van Heerwaarden et al., 2024). Further work such as pedigree crosses can determine the mechanisms underlying this failure of reproduction at high temperatures. For examples, sperm viability, ovariole development or energetic investment into the eggs are temperature sensitive (Kindlmann et al., 2001; Steigenga and Fischer, 2007). Whether mating behaviours are influenced by developmental temperature can be important to know for insect farms with separate breeding and rearing environments.

Our data suggests there are few costs or trade-offs of living at a high temperature among the traits we characterised provided temperatures are below the thermal limit of survival. An absence of a trade-off could arise if life history traits have different nutritional requirements, or if *ad libitum* conditions, including the presence of conspecifics, free up individuals from allocating limited resources in a trade-off, which would explain also the absence of an optimal temperature (Shoemaker and Adamo, 2007; Roark and Bjorndal, 2014). Testing these relationships by scaling up to group-reared insects is crucial as context-specific variables can emerge in group-rearing setting but evidence for potential effects of density is mixed. Growth differs between group-reared (< 500 individuals) and individually reared *G. sigillatus*, but these values do not approach the densities used for commercial rearing, and rearing density has no effect in other farmed insects (Muzzatti et al., 2025; Zim et al., 2022). Indirectly, group-rearing allows for cannibalism which may benefit individuals that can cannibalise more (Hansen et al., 2011). If temperature directly or indirectly influences cannibalism rates, then group survival and yield will be impacted even if individual fitness is maximised. Density and the social environment may strongly influence population dynamics at the very high densities used for commercial rearing.

Rearing practices at a farm will determine the selection pressures of the farmed population, which raises several unique inferences for insect mass-rearing compared to natural populations. If crickets are harvested after a breeding period at the source population then breeding and production come from the same population with a unique and intense selection pressure that can affect several life history traits (Hanboonsong and Durst, 2020). First, the selection pressure of a complete harvest may result in an absence of sex-specific differences. Late developing crickets would not be able to reproduce before harvest and individual fitness is maximised for crickets that can reproduce earlier. Second, body, spermatophylax and sperm ampulla mass, as well as lifespan, mortality and adult ageing are highly heritable in *G. sigillatus* (Gershman et al., 2010; Archer et al., 2012). These high heritabilities may facilitate the similar lifespans between males and females, because these traits could be advantageous for a mass-reared population under the strong selection pressure imposed by a complete harvest. Larger sizes correspond with higher reproductive fitness in male and female crickets, and we found larger females were more fecund regardless of rearing temperature (Wedell, 1997; Simmons and García-González, 2007). Third, harvest schedules may impose a stabilising selection pressure on the relationship between development rate and longevity. Crickets that develop rapidly but also die naturally before a harvest and crickets that develop slowly but live longer will have lower fitness than crickets that can develop rapidly but survive until the harvest. Separating a breeding population from the production population would alleviate the selection pressures imposed by harvesting and may allow for better control against inbreeding. Inbreeding depression could explain the lower fecundity of our population compared with other studies as *G. sigillatus* exhibits strong inbreeding depression, but we have yet to characterise genetic diversity in our population (Sakaluk et al., 2019). Our current knowledge on *G. sigillatus* suggest this species is highly evolvable which would explain why they have a global tropical distribution with a close association with human environments (Bateman et al., 2005; Archer et al., 2012). A direct comparison of phenotypes and genotypes of farmed and wild crickets would allow us to identify the nature of selection during the farming process, identify potential inbreeding or bottlenecks, and suggest ways to improve current farmed strains.

Our conclusions for optimising rearing temperatures differ between considering individual traits and a calculated yield index. We found that the influence of temperature on traits relevant to yield were similarly impacted by temperature but to varying degrees. There was therefore no single optimum temperature suitable for all traits (Clark et al., 2013; Blanckenhorn et al., 2021). An optimal temperature for rearing was better represented by a range of temperatures that maximised biomass and minimised rearing time within the time constraints of a harvest (Abarca et al., 2024). Based on a six-week harvest schedule, development rate to adulthood sets the lower temperature limit (30 °C). Longevity and reproduction set the upper temperature limit (38 °C). Our conclusions based on individual traits suggests *G. sigillatus* life history traits are not tightly linked through thermal plasticity and crickets can tolerate a range of temperatures without significant variation in performance (Kellermann et al., 2019). Thus, precise temperature control around a single optimum is unnecessary for rearing *G. sigillatus* and varying temperatures between 32 and 36 °C are not expected to significantly affect performance directly. The effect of varying temperature should be tested in production. Farms can also explore the potential for optimising temperature for a specific purpose or life stage during rearing (Li et al., 2023).

In contrast, our estimations of yield suggest an optimal temperature exists if maximising yield is a priority for rearing. Calculated yield did not significantly differ within the optimal temperature range for rearing (32–36 °C), but the highest potential yields were calculated at 36 °C (Fig. 3A). Growth and development limits yield below 30 °C as these crickets had not reached maturity (Fig. 3B). Survival would likely limit yield at 20 and 22 °C because survival trended downwards rapidly at these temperatures in the first eight weeks (Fig. 1A). Our weekly yield estimates suggest rearing at 32 and 34 °C would produce similar yields if harvested at the same time. Our calculations also suggest rearing at 36 °C (the limit of rearing) would allow for harvest a week earlier compared with 34 °C, but this difference may also be risky for an insect farm because the decline in yield between 36 and 38 °C is large (Fig. 3B). The proximity to a potential decline in performance is significant for mass-rearing because additional heat generation from individuals or from microbial respiration in high-density population would modify the effective temperature for insects even under temperature-controlled environments (Li et al., 2023). Thus, increasing rearing temperature may have diminishing return on yield, which affects the cost-benefit of a high rearing temperature.

Predicted yield allowed us to assess the combined effects of several life history traits which may be a more informative way of assessing thermal performance than individual traits (Muzzatti et al., 2024). For example, trait values measured late in development only represent individuals that survived a temperature treatment and thus may be biased against temperatures approaching the limits of performance (Abarca et al., 2024). We used an individual-level metric of yield based on development time and mass because our data is at the scale of the individual. However, our calculations do not account for differences in survival to adulthood (calculated for the population) or sex, meaning our values are biased towards crickets that survived, nor for variation in longevity. Survival will also affect the potential population-level reproductive output, which has consequences for maintaining a farmed population that is underestimated by our individual-level data on reproductive output. A population-level yield metric would account for survival, but our study lacks the required replication. Survival is implicitly captured by yield as exemplified by crickets at 38 °C that had a similar yield to 34 and 36 °C for the first three weeks but lower yield than the other temperatures at later weeks because the crickets were rapidly dying. Differences in survival between sexes also explains why yield at 38 °C did not show a similar decrease during weeks 5–7 seen for 30–36 °C crickets. Larger scale group-rearing studies likely benefit from measuring yield directly (population-level total biomass).

An area of future work could investigate the mechanisms that mediate thermal plasticity in traits. We did not measure any potential physiological mechanisms that may mediate life history trade-offs at the whole-organism level, such as bioenergetics, microbiomes, or immunity (Jordan and Tomberlin, 2021; Vogel et al., 2022). If temperature mediates an energetic trade-off, then measuring metabolic rates of crickets reared at different temperatures should provide a baseline for the energetic cost associated with our observed life history traits (Lachenicht et al., 2010; Okada et al., 2011). These data would allow us to calculate the cost of development and identify temperatures that impose a lower energetic cost for growth (Marshall et al., 2020). Temperature-dependent energy allocation to growth may also be influenced by genetics or diet, but temperature and diet may act to shape life history independently of metabolic rate (Chakraborty et al., 2023; Alton et al., 2024). Future work exploring diet and temperature interactions will be important in developing optimised rearing protocols for farmed insects.

Our data form a fundamental knowledge base to direct future studies and evaluate current farming practice to maximise the yield of a specific species. An important step forward in developing this knowledge base for *G. sigillatus* is to characterise temperature-dependent development for the complete life cycle, including the egg stage, as well as investigate other abiotic conditions such as humidity and photoperiod and how these abiotic conditions interact. Notably, we had drastically lower survival at 38 °C in the reproduction experiment (30 %) compared with the life history experiment (67 %). This difference is most likely because of the lower relative humidity in the incubator (50 %) in combination with the high temperature compared with the life history experiment (64 %) that contributed towards high early life mortality in the first five days of the experiment. Assessing performance traits across the whole life cycle, such as egg-to-adult survival, may reveal specific life stages that represent vulnerable bottlenecks, and a single life stage may not be representative of vulnerabilities throughout the life cycle (Amarasekare and Sifuentes, 2012; Rebolledo et al., 2020). Overall, we can ensure traits that are favourable for mass-rearing are present in farmed populations and these relationships can be used throughout the supply chain to optimise the rearing and processing of insects for industrial use (Sinclair et al., 2022). Another important step is to understand how our results inferred from individuals would scale up to an insect farm. Understanding this relationship requires manipulating rearing temperature under mass-rearing conditions, and in partnership with a mass-rearing facility that can concurrently evaluate the operative costs and benefits of manipulating temperature. Notably, *G. sigillatus* is a tropical species that is mass-reared indoors in temperate areas, such as North America, as opposed to well-ventilated rearing sheds suggested for rearing crickets in tropical regions, such as Southeast Asia (Hanboonsong and Durst, 2020). The feasibility of outdoor rearing depends on matching the thermal performance of species to environmental temperatures, whereas indoor rearing requires heating which contributes towards the resource use and carbon footprint of insect farming (Magara et al. 2024; van Huis and Oonincx, 2017). Energy use (gas and electricity) is estimated to contribute up to 43 % of greenhouse gas emissions from mealworm mass-rearing (Oonincx and de Boer, 2012). Similarly, food consumption increases with rearing temperature in crickets, which will affect the operational costs of rearing (Morales-Ramos et al., 2018). Therefore, any benefits of a high rearing temperature need to be considered against potential environmental and economic costs for industrial mass-rearing.

## CRediT statement

**Jacinta Kong:** Conceptualization, Methodology, Investigation, Formal analysis, Data Curation, Visualization, Writing - Original Draft. **Émile Vadboncoeur:** Methodology, Investigation, Writing - Original Draft. **Susan Bertram:** Conceptualization, Supervision, Funding acquisition. **Heath MacMillan:** Conceptualization, Supervision, Funding acquisition. All authors contributed towards Writing - Review & Editing.

## Data availability

Data generated or analysed during this study are available at Zenodo https://doi.org/10.5281/zenodo.14681072.

## Declaration of competing interest

The authors have ongoing research agreements with Aspire Food Group, Entomo Farms, and Bug Mars, which are all companies operating the in the insects as food and feed sector.
